# Epithelial cell-directed efferocytosis in the post-partum mammary gland is necessary for tissue homeostasis and future lactation

**DOI:** 10.1186/1471-213X-10-122

**Published:** 2010-12-30

**Authors:** Melissa Sandahl, Debra M Hunter, Karen E Strunk, H Shelton Earp, Rebecca S Cook

**Affiliations:** 1 UNC-Lineberger Comprehensive Cancer Center, 450 West Ave, Chapel Hill, North Carolina 27599, USA; 2 Department of Medicine, University of North Carolina School of Medicine, 450 West Ave, Chapel Hill, North Carolina 27599, USA; 3 Department of Pharmacology, University of North Carolina School of Medicine, 450 West Ave, Chapel Hill, North Carolina 27599, USA; 4 Department of Cancer Biology, Vanderbilt University School of Medicine, 2220 Pierce Ave., Nashville, TN 37232, USA; 5 The Vanderbilt-Ingram Cancer Center, Vanderbilt University Medical Center, 2220 Pierce Ave., Nashville, TN 37232, USA

## Abstract

**Background:**

Mammary glands harbor a profound burden of apoptotic cells (ACs) during post-lactational involution, but little is known regarding mechanisms by which ACs are cleared from the mammary gland, or consequences if this process is interrupted. We investigated AC clearance, also termed efferocytosis, during post-lactational remodeling, using mice deficient for MerTK, Axl, and Tyro3, three related receptor tyrosine kinases (RTKs) regulating macrophage-mediated efferocytosis in monocytes. MerTK expression, apoptosis and the accumulation of apoptotic debris were examined in histological sections of MerTK-deficient, Axl/Tyro3-deficient, and wild-type mammary glands harvested at specific time points during lactation and synchronized involution. The ability of primary mammary epithelial cells (MECs) to engulf ACs was assessed in culture. Transplant of MerTK-deficient mammary epithelium into cleared WT mammary fat pads was used to assess the contribution of WT mammary macrophages to post-lactational efferocytosis.

**Results:**

ACs induced MerTK expression in MECs, resulting in elevated MerTK levels at the earliest stages of involution. Loss of MerTK resulted in AC accumulation in post-lactational MerTK-deficient mammary glands, but not in Axl and Tyro3-deficient mammary glands. Increased vascularization, fibrosis, and epithelial hyperproliferation were observed in MerTK-deficient mammary glands through at least 60 days post-weaning, due to failed efferocytosis after lactation, but did not manifest in nulliparous mice. WT host-derived macrophages failed to rescue efferocytosis in transplanted MerTK-deficient mammary epithelium.

**Conclusion:**

Efferocytosis by MECs through MerTK is crucial for mammary gland homeostasis and function during the post-lactational period. Efferocytosis by MECs thus limits pathologic consequences associated with the apoptotic load following lactation.

## Background

The stromal microenvironment in which the breast epithelium exists greatly influences its physiology, function, and architecture [1-3]. In the mammary gland, the stroma is comprised of fibroblasts, adipocytes, vessels, lymphatics, and immune effector cells. Tightly regulated epithelial-stromal interactions direct every aspect of pre-and post-natal mammary gland development, including the profound changes that occur during pregnancy, lactation, and involution. Specifically, macrophages in the mammary gland contribute to epithelial growth during puberty, epithelial differentiation during pregnancy, and are recruited to the mammary gland during involution [3-5]. Alterations in the stromal macrophage population can have pathologic consequences on the development and maintenance of the mammary gland [6]. For example, mice harboring a null mutation in the *Csf1 *gene (*Csf1^op^*) have decreased numbers of mammary macrophages, resulting in reduced terminal end bud numbers, branching, and ductal elongation in the mammary epithelium [6-8]. Following pregnancy these mice fail to nurse their pups due to inadequate differentiation of the mammary epithelium and poor milk supply [9].

Apoptosis occurs at some level in most tissues, and is a critical aspect of tissue development, lymphocyte maturation, and normal cell turnover. Clearance of apoptotic debris is required to prevent secondary necrosis, chronic inflammation, release of self molecules, and tissue damage. Released self-molecules may become antigenic and cause lymphocyte activation and autoantibody production [10]. In the mammary gland, apoptosis allows for the plasticity that characterizes the many stages of mammary gland biology. Apoptosis occurs during puberty, canalizing the solid epithelial cords that form the ductal epithelium [11]. With each menstrual cycle in humans (or the estrous cycle in mice), the mammary epithelium undergoes modest proliferation of lobuloalveolar buds, which either continue proliferating in the event of pregnancy, or otherwise undergo apoptosis [12]. Following pregnancy and lactation, the mammary epithelium undergoes involution, culling the vast majority (up to 90%) of the mammary epithelium within only 7-10 days, leaving a relatively quiescent ductal epithelial tree [3,4].

The profound burden of ACs during post-lactational involution necessitates a mechanism for the rapid clearance of these cells. Also for normal mammary gland remodeling to take place, milk fat globules and residual milk must also be efficiently and rapidly removed. The debate regarding the removal of ACs in the mammary gland as a job of resident mammary macrophage or of neighboring MECs has support from both sides [1,2,4,6]. In the mammary gland, macrophage infiltration is detected between days 2 and 4, at which time they have been shown to engulf dying MECs and to release anti-inflammatory cytokines, such as TGFβ1. However, comprehensive analyses demonstrated that apoptotic mammary epithelial cells are apparent and become cleared within hours of removing pups from a nursing dam [13], prior to macrophage infiltration of the involution mammary gland. In cell culture, primary cells and established cell lines from the mouse mammary epithelium were capable of binding and engulfing ACs, albeit much less efficiently than macrophages. While these studies did not identify which signaling pathways were necessary for AC clearance by MECs, it was found that some of the receptors used by macrophages for AC clearance were also expressed by MECs during involution, including the phosphatidyl serine receptor, integrin α_v_β_3 _, calreticulin, and CD91 [3,13].

To understand the role of AC clearance in the post-lactational mammary gland *in vivo*, we utilized a genetically engineered mouse model that harbors macrophages with an impaired ability to engulf ACs. This model lacks the receptor tyrosine kinase MerTK, a member of the TAM (Tyro-3/Axl/MerTK) family [14-17]. Each member of the TAM receptor family shares structural similarity in the extracellular region and a hallmark KWAIAES motif in the cytoplasmic kinase domain [18]. Each of the TAM receptors is involved in AC clearance by macrophages and/or dendritic cells [18-22]. MerTK is required for AC clearance by macrophages, and for the subsequent dampening of inflammatory cytokine secretion in order to limit potentially destructive immune responses [20,23-26]. *mertk^-/- ^*macrophages and DCs secrete pathologically high levels of tumor necrosis factor-α (TNF-α) in response to lipopolysaccharide, demonstrating the role of MerTK in dampening acute inflammatory responses in macrophages [22,27].

Very little is known regarding TAM family RTKs in mammary gland development, neither in regard to epithelial autonomous effects nor those produced by interactions between the developing mammary epithelium and resident macrophages. We show herein that growth and branching of the ductal epithelium during puberty occurs unabated in the absence of MerTK, as does expansion and differentiation of the secretory epithelium. In the MerTK-deficient model, we found a striking accumulation of ACs and stagnant milk in ductal lumens during post-lactational involution, resulting in stromal and epithelial alterations that were sustained through at least 60 days of involution. While apoptotic debris was eventually cleared in the absence of MerTK, mammary glands from *mertk^-/- ^*mice displayed persistent hyperproliferation, increased vascularization, and fibrosis. Phenotypic changes in MerTK-deficient mammary glands were evident by day 3 of involution, prior to the peak of macrophage infiltration previously described [3,5]. Surprisingly, we found that MerTK expression was elevated in the mammary epithelium at involution day 1, and that while WT MECs were capable of engulfing ACs *ex vivo*, *mertk^-/- ^*MECs were not. Transplantation of *mertk^-/- ^*mammary epithelium into WT mammary fat pads resulted in AC accumulation, and post-lactational increases in epithelial cell proliferation, stromal vascularization, and fibrosis, despite the presence of WT macrophages derived from the WT host environment. These studies demonstrate that AC clearance during post-lactational involution is performed primarily by neighboring MECs, that this process requires MerTK, and that the consequences of failed AC clearance during post-lactational involution result in sustained alterations in the stroma and epithelium that prevent subsequent lactation.

## Methods

### Mice

All mice regardless of genotype were inbred to C57Bl/6J for at least 10 generations. *mertk^-/- ^*mice, originally referred to as Mer^KD ^mice; have been previously described [27]. To generate WT and *mertk^-/- ^*siblings, *mertk^+/- ^*males and females were mated to generate the expected Mendelian ratios of WT, *mertk^+/-^*, and *mertk^-/- ^*offspring. *Tyro3^-/-^*/*Axl^-/-^*mice have been described previously [27], and were gifts from the laboratory of Dr. Glenn Matsushima, University of North Carolina, Chapel Hill, NC. Actin-GFP transgenic mice were from Jackson Laboratories (Bar Harbor, ME). Mice were maintained in AAALAC approved animal facilities using protocols approved by the University of North Carolina Institutional Animal Care and Use Committee.

### Establishing time points of pregnancy, lactation, and involution

For timed pregnancies, mating pairs were established and mice were monitored daily for vaginal plugs, indicating 0.5 d.p.c. The day of parturition was considered lactation day 1. For lactation time points, mammary glands were analyzed at lactation day 10 (or lactation day 1 for mammary transplants and some second pregnancies). To synchronize involution in each of the 10 mammary glands per mouse, pups were withdrawn at lactation day 10 (or lactation day 1 for mammary transplants), indicating 0 days post-forced wean (dpfw). Mammary glands were examined at 3, 7, 10, 14, 21, and 60 dpfw.

### Histological analysis and immunohistochemistry (IHC)

Mammary glands were harvested and immediately fixed in 10% formalin (VWR Scientific). Hematoxylin-stained whole mounts of #4 (right inguinal) mammary glands were prepared as previously described [28]. Paraffin-embedded mammary glands were sectioned (5 μm), rehydrated, and peroxidase was quenched with 3% H_2_O_2_. IHC was performed as previously described [29] using the following polyclonal antibodies: MerTK (synthesized in this laboratory, against a peptide in the extracellular domain of human MerTK); collagen IV (1:200, Santa Cruz Biotechnologies), PECAM/CD31 (1:100, Santa Cruz Biotechnologies). Slides were washed in PBST then incubated in biotinylated anti-rabbit antibody (Vector Laboratories, Burlingame, VT) and developed using the Vectastain Kit (Vector Laboratories). Immunohistochemistry for PCNA was performed using the PCNA Staining Kit (Zymed) according to manufacturer's instructions. TUNEL analysis was performed using the ApopTag In Situ Detection Kit (Promega, Madison, WI). Cells were photographed using the Zeiss LCM 210 microscope and Scion Image 2.0 software.

### Transplantation of mammary tissue into cleared mammary fat pads

Donor mammary tissue was harvested from the inguinal mammary glands (lymph nodes removed) of female WT and *mertk^-/- ^*mice at 6 weeks of age, under sterile conditions. Genotype of the donor mammary tissue was confirmed by PCR of genomic DNA. Recipient female mice were 3 week-old WT C57Bl/6 mice. The right and left inguinal mammary fat pads of recipient females were cleared of endogenous epithelium by surgical removal of the proximal one-third of the mammary fat pad, in which the epithelium is exclusively located in 3 week-old mice. A single 2 mm^3 ^portion of mammary tissue from donor mice was surgically placed into the cleared mammary fat pad of recipient mice. Each recipient mouse received WT mammary tissue in the right cleared fat pad, and *mertk^-/- ^*mammary tissue in the left cleared fat pad, allowing the development of the reconstituted mammary glands to be internally controlled and examined as a matched pair. Eight weeks following transplant mice were bred in order to examine the mammary glands during specific stages of involution as described above.

### qRT-PCR

Total mammary RNA was harvested from flash-frozen mammary glands taken from mice at 10 dpfw, or from cultured cells using the RNeasy kit (Qiagen). Total RNA (10 ng) was reverse transcribed with transcript-specific primers, then amplified with transcript-specific primers in the presence of a TAM-labeled, target-specific probe, using the AmpliTaq EZ RT-PCR kit (ABI), according to manufacturer's instructions, with a Tm of 62°C. The following primers and probes were used: mouse MerTK: Forward 5'-TGA CTC CTT GGA AGA CTC TGA AG; reverse 5'-CTT GAA GAT TGC CTT GGT CAT; probe 5'-GTG GTC TTA GAT ACT TTG TTA; and mouse tgfb1. The CT for each transcript within each sample was corrected for the CT of *gapdh *within each sample, then normalized to the CT of a single sample, and converted into relative level of expression using the δδCT method, such that all values are presented as a fold change in reference to a single sample. Samples were analyzed six times.

### Isolation and culture of primary mammary epithelial cells (PMECs)

Mammary glands were collected under aseptic conditions and dispersed in 1X collagenase-hyaluronidase solution (Stem Cell Technologies, Vancouver, British Columbia, Canada) overnight at 37°C, digested briefly with trypsin, then washed and plated at a density of 500,000 cells per 35-mm dish in DMEM-F12 supplemented with 2% FBS, 10 ng/ml EGF, 5 μg/ml insulin, and 5 μg/ml dexamethasone. Where indicated, media was supplemented with 25 μg/ml recombinant Gas6 (R&D Systems, Rockville, MD). Cells were co-cultured with 2.5 × 10^6 ^apoptotic thymocytes harvested from dexamethasone-treated Actin-GFP female mice. To harvest thymocytes, whole thymus was collected from mice then physically dissociated through a 70 μm mesh filter in phosphate buffered saline pH 7.4 (PBS). Red blood cells were lysed with sterile deionized water, and remaining thymocytes were washed twice with PBS. Apoptotic thymocytes were counted and plated with adherent PMECs at a ratio of 5:1 (apoptotic thymocytes to PMECs). Cells were co-cultured in the presence of Gas6 for 4 hours, then PMECs were washed 5 times with PBS to remove non-engulfed thymocytes. PMECs were collected by trypsinization and analyzed for GFP incorporation by flow cytometry at the UNC-Lineberger Comprehensive Cancer Center Flow Cytometry Core Facility.

## Results

### Accumulation of ACs in post-lactational *mertk^-/- ^*mammary glands

**Figure 1:**
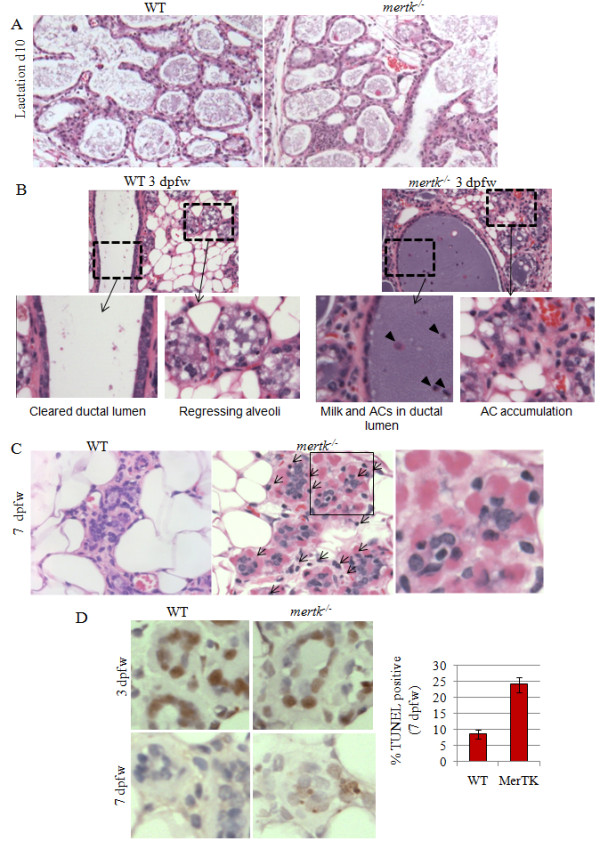
**Impaired clearance of apoptotic mammary epithelial cells during involution in MerTK-deficient mice**. **A**. H&E-stained sections of mammary glands harvested at lactation day 10 from WT or *mertk^-/- ^*mammary glands. **B-C**. H&E-stained sections of mammary glands harvested at 3 dpfw (**B**) and 7 dpfw (**C**) from WT and *mertk^-/- ^*mammary glands. Boxed areas are shown in higher magnification, where indicated in B indicate AC accumulation in the ductal lumen and in regressing alveolar structures. Arrows in C indicate apoptotic cells surrounding live cells in a rosette pattern. **D **Immunohistochemical detection of TUNEL-positive cells at 7 dpfw. Values shown in D (left panel) are the average percentage of total nuclei that are TUNEL-positive. N = 5 (X 3 random 400X fields per sample). P = 0.002, Student's unpaired T-test.

To investigate the role of MerTK in AC clearance during involution, pregnancies were established in 12-week old virgin female mice. Loss of MerTK did not alter the number of pups per litter (an average of 5.4 versus 5.8 pups per litter to born to *mertk^-/- ^*and WT dams, N = 10 dams per genotype). Lactation by *mertk^-/- ^*females was sufficient to support the nursing pups. Histological evaluation of *mertk^-/- ^*mammary glands at lactation day 10 (L10) was unremarkable (Figure 1A). Pups were withdrawn at L10 to force synchronized involution of the mammary glands. Collapsed secretory structures and reappearance of adipocytes was evidence that milk production had terminated by 3 dpfw in wild-type and MerTK-deficient samples. While the ductal lumens of WT samples were clear at 3 dpfw, milk and ACs filled the lumens of *mertk^-/- ^*samples (Figure 1B). Residual apoptotic cells remained evident at 7 dpfw in MerTK-deficient samples (Figure 1C), appearing in rosette structures surrounding live cells. TUNEL analysis confirmed the presence of apoptotic cells at 7 dpfw in MerTK-deficient samples but not in WT samples (Figure 1D). These data are consistent with the role of MerTK in macrophage-mediated efferocytosis of ACs, and suggest that MerTK is required for efferocytosis during involution.

### Impaired efferocytosis during involution causes chronic post-partum stromal activation with increased vascularization, fibrosis, and epithelial proliferation

**Figure 2:**
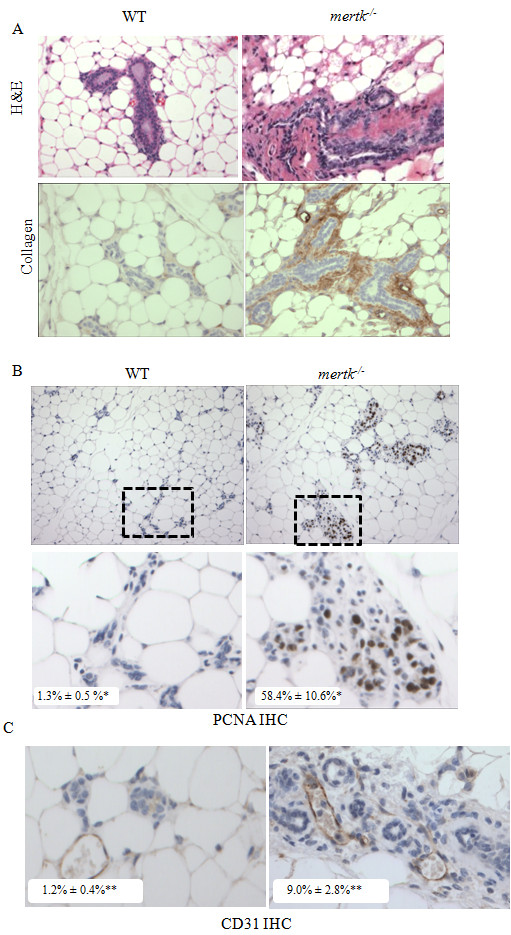
**Impaired apoptotic cell clearance results in pathological changes that are sustained through 60 dpfw**. **A**. Sections of mammary glands harvested at 60 dpfw were stained with H&E and with an antibody against collagen. **B**. Immunohistochemical detection of PCNA in mammary glands harvested at 60 dpfw. N = 5 per genotype. Values shown in bottom left of each panel represent the average percentage of total nuclei per 400X field (± S.D.) that were PCNA+. *P = 0.045; Student's unpaired T-test, N = 3 samples per group, 5 fields per sample. **C**. Immunohistochemical detection of the blood vessel marker PECAM/CD31. Values shown in the bottom left of each panel represent the average number of vCD31-positive vessels per 400X field (± S.D.). **P = 0.0001. Student's unpaired T-test, N = 3 samples per group, 5 fields per sample.

In WT mammary glands at 60 dpfw, the lobuloalveolar epithelium had regressed completely, such that adipose tissue was sparsely populated with narrow ductal structures surrounded by minimal extracellular matrix (Figure 2A). In the *mertk^-/- ^*mice, periductal ECM accumulation was evident, accompanied by increased stromal cellularity. Immunohistochemical detection of collagen illustrated the accumulation of collagen in the periductal areas of *mertk^-/- ^*mammary glands. Immunohistochemical detection of proliferating cell nuclear antigen (PCNA) was used to measure cellular proliferation *in situ*, revealing a higher proportion of PCNA+ epithelial cells in MerTK-deficient samples as compared to WT (Figure 2B). Immunohistochemical detection of the endothelial cell marker CD31 demonstrated increased vessel number in MerTK-deficient mammary glands versus WT (Figure 2C).

### Pathological consequences of impaired MerTK-induced efferocytosis are restricted to the post-partum mammary gland

**Figure 3:**
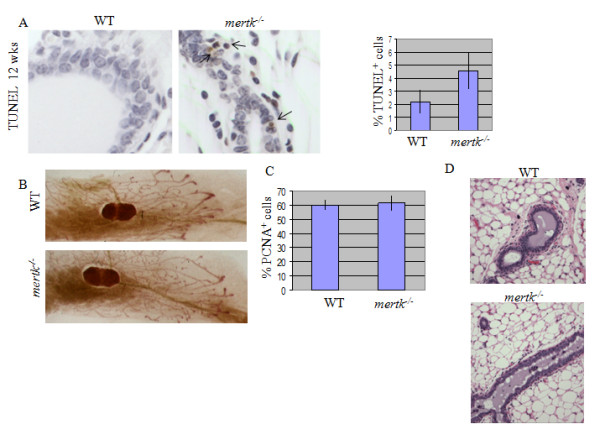
**Pathologic consequences of impaired apoptotic cell clearance are specific to the post-partum mammary gland**. **A**. Increased presence of apoptotic cells in MerTK-deficient mice was shown by TUNEL analysis of mammary glands from 12-week old virgin female mice. Values represent the average percentage of the total epithelial population that was TUNEL-positive, ± S.D. N = 3 (5 fields per sample). P = 0.01, Student's T-test. **B**. Whole mounted, hematoxylin-stained mammary glands harvested from eight week old mice. C. mammary glands from 12-wekk old virgin female mice were analyzed by immunohistochemistry for PCNA. Values shown represent the average percentage of total epithelial nuclei that were PCNA-positive, ± S.D. P = 0.39, Student's T-test. **D**. H&E-stained sections from WT and *mertk^-/- ^*mammary glands harvested from virgin mice at 12 weeks of age.

While ACs were detected at two-fold higher levels in virgin MerTK-deficient mice as compared to virgin WT mice (Figure 3A), growth of the ductal epithelium during puberty was not affected by loss of MerTK as determined by whole mount hematoxylin staining of mammary glands from 8 week old virgin female mice (Figure 3B), and by immunohistochemical detection of PCNA (Figure 3C). Unlike MerTK-deficient mammary glands harvested at time points post-partum, mammary glands from virgin MerTK did not display peri-ductal ECM accumulation, increased vascularization, or epithelial hyperplasia (Figure 3D). Therefore, phenotypic effects of MerTK-deficiency manifested after a single round of pregnancy, lactation, and involution, and were therefore the result of the post-partum environment. Because the pathologies evident in *mertk^-/- ^*mammary persisted through at least 60 dpfw, this suggests that the increased epithelial content observed in *mertk^-/- ^*mammary glands was not due to delayed involution, since the phenotype did not resolve even over extended time points.

### Axl and Tyro3 are not required in the mammary gland to achieve homeostasis following lactation

**Figure 4:**
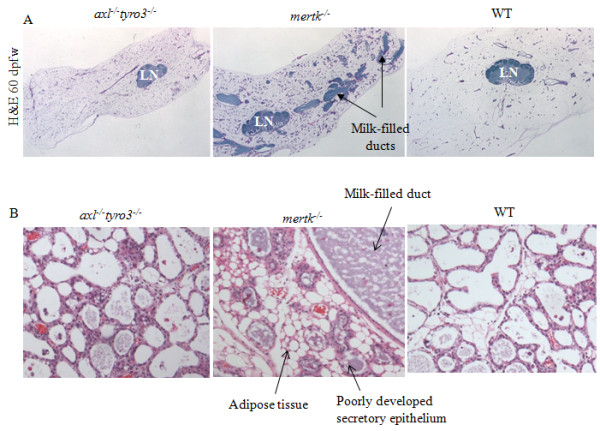
**Loss of MerTK, but not Axl or Tyro3, causes post-lactational pathologies that prevent lactation following second pregnancy**. **A**. Low-power magnification of H&E-stained section of WT, *mertk^-/- ^*and *Axl^-/-^Tyro3^-/- ^*mammary glands harvested at 60 dpfw, demonstrating the tissue-wide extent of the pathological changes occurring in MerTK-deficient samples. Arrows indicate milk-filled distended lumens in *mertk^-/- ^*samples. B. At 60 dpfw, mice were impregnated for a second time. Mammary glands shown here were harvested at lactation day 1. Note the incomplete development of the lobuloalveolar epithelium in MerTK-deficient samples.

In addition to MerTK, the other members of the TAM family, Axl and Tyro3, also have known roles in AC clearance by macrophages and DCs, and subsequent cytokine modulation [22,30,31]. To determine if loss of Axl and Tyro3 results in similar post-partum changes in the mammary gland, we examined the mammary glands of Axl/Tyro3 double-deficient mice at 60 dpfw (after a single pregnancy and lactation). Similar to what was seen in WT samples, the epithelium of *axl^-/- ^X tyro3^-/- ^*mammary glands was sparse, and the adipose tissue was abundant at 60 dpfw (Figure 4A). No milk accumulation was seen in WT or *axl^-/- ^X tyro3^-/- ^*mammary glands at 60 dpfw. In contrast, static milk persisted in the dramatically distended ducts of *mertk^-/- ^*samples, and epithelial structures appeared more prominent.

After a single round of pregnancy, lactation, and 60 days of involution, mice were impregnated for a second time to determine the impact of post-lactational efferocytosis on the ability to nurse subsequent litters. It was found that 8/8 WT dams and 8/8 *axl^-/- ^X tyro3^-/- ^*dams were able to nurse second litters successfully after 60 days of involution. In contrast, only 4/8 MerTK-deficient dams supported litters through weaning. Histological examination of mammary glands at second lactation day 1 revealed that, while WT and *axl^-/- ^X tyro3^-/- ^*mammary glands were encompassed by fully developed secretory epithelium, large regions of MerTK-deficient mammary glands harbored poorly developed epithelial structures, such that adipose tissue was widely visible (Figure 4B). These data underscore the role of MerTK-directed efferocytosis in mammary gland function and homeostasis. Furthermore, these results suggest that efferocytosis in the mammary gland relies on MerTK, but not other members of the TAM family of receptors.

### WT macrophages do not compensate for the loss of MerTK in the mammary epithelium, resulting in apoptotic cell accumulation

Because phenotypic aberration due to impaired efferocytosis were limited to MerTK-deficient mammary glands, but all TAM receptors regulate macrophage-mediated efferocytosis, we speculated that non-professional phagocytes within the mammary gland may be responsible for clearance of apoptotic cells during the post-lactational period. Recent evidence suggests that the mammary epithelium may be responsible for the clearance of apoptotic neighboring MECs [5,13]. To distinguish between AC clearance mediated by macrophages versus by mammary epithelium, we reconstituted cleared inguinal mammary fat pads of WT mice with both WT and *mertk^-/- ^*mammary tissue. Each recipient WT mouse received *mertk^-/- ^*mammary transplant in the left mammary fat pad, and WT mammary transplant into the right mammary fat pad. Eight weeks following transplant, recipients were impregnated. Pups were withdrawn at birth to initiate synchronized involution of all mammary glands. Mammary glands were analyzed at 14 dpfw.

**Figure 5:**
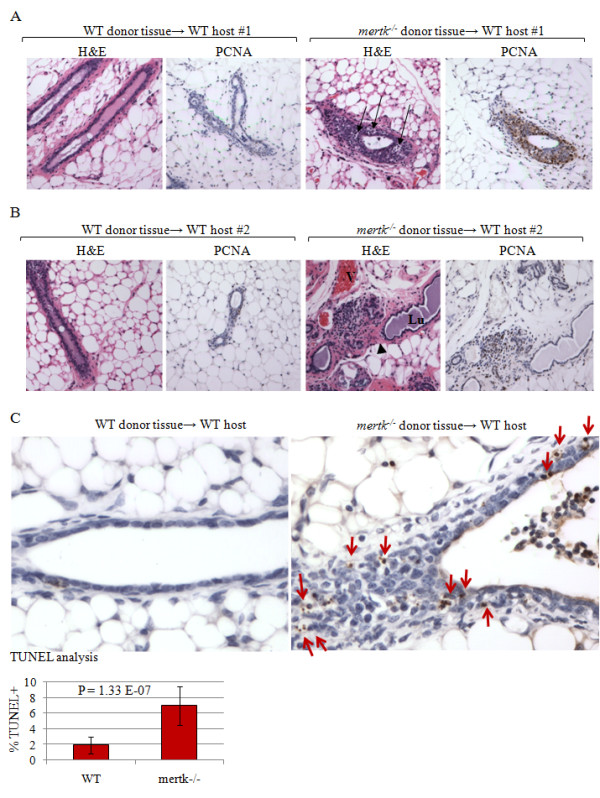
**WT macrophages do not compensate for impaired efferocytosis by MerTK-deficient mammary epithelium**. **A-B**. Histological examination of WT mammary glands cleared of endogenous epithelium and central lymph nodes and reconstituted with either WT or MerTK-deficient mammary epithelium. For each host mouse, WT tissue was transplanted into the right #4 fat pad, and *mertk^-/- ^*tissue was transplanted into the left #4 fat pad. Paired sets are displayed. Mice were impregnated 8 weeks following mammary transplant, pups were withdrawn at lactation day 1 to enforce involution, and mammary glands were harvested at 14 dpfw. H&E images and PCNA immunohistochemistry are shown. **A**. Images taken of the right and left reconstituted mammary glands from host female #1. **B**. Images taken of the right and left reconstituted mammary glands from host female #2. **C**. TUNEL analysis of paired reconstituted mammary samples from a single WT recipient mouse, showing the mammary gland reconstituted with WT mammary epithelium and *mertk^-/- ^*mammary epithelium. Arrows indicate apoptotic bodies. Values represent the average percentage of the total epithelial nuclei that were TUNEL-positive, ± S.D. Significance calculated using Student's paired T-test. N = 7.

WT and *mertk^-/- ^*mammary epithelium resulted in successful epithelial repopulation of the cleared fat pads (Figure 5A and 5B). While WT mammary epithelium produced single-layer ductal epithelium, limited peri-ductal stromal activation, and few PCNA+ cells, *mertk^-/- ^*mammary epithelium within the WT fat pad was multi-layered, was surrounded by a thickened peri-ductal stroma with abundant stromal cellularity, and displayed elevated PCNA+ cells in epithelial and stromal cell layers. These findings were recapitulated in 100% (N = 7) of paired samples examined. TUNEL analysis demonstrated accumulation of ACs in *mertk^-/- ^*mammary epithelium as compared to the WT epithelium (Figure 5C). Because both of these epithelial cell populations developed within an identical stromal environment, this data confirms that accumulation of apoptotic MECs in the absence of MerTK is a phenotype that is epithelial autonomous. These data do not rule out the contribution of mammary macrophages in AC clearance. However, because WT professional phagocytes from the host should be equally available to both WT and *mertk^-/- ^*MEC populations, These data suggest that ACs in the post-partum mammary gland are cleared primarily by neighboring MECs, and not by professional phagocytes.

### MerTK is required for ingestion of apoptotic cells by mammary epithelial cells

**Figure 6:**
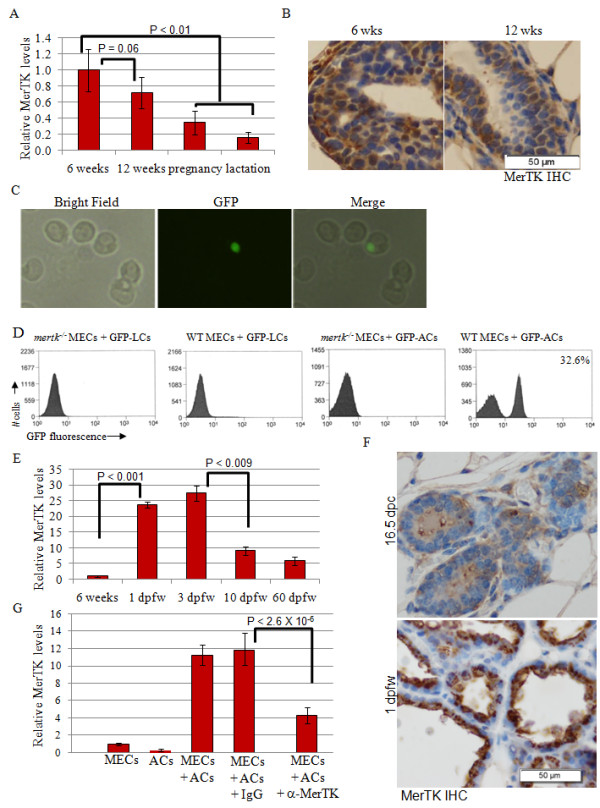
**MerTK directs efferocytosis in mammary epithelial cells**. **A**. Taqman™ real-time PCR was used to quantitate the relative levels of *mertk *mRNA in mouse mammary total RNA, harvested at the indicated time points. The relative levels of *mertk *were calculated using expression of mouse GAPDH to correct for total RNA concentration using the δδCT method, setting the value of *mertk *expression measured at 6 weeks to 1. Average values are shown ± S.D. N = 3, each sample analyzed 6 times. Statistical significance using Student's T-test (unpaired). **B**. Immunohistochemistry to detect MerTK in WT mammary glands harvested from virgin female mice at 6 and 12 weeks of age. Representative images are shown. **C**. WT and *mertk^-/- ^*PMECs were incubated for 4 hours in the presence of live or apoptotic GFP+ cells harvested from the thymus of untreated or dexamethasone-treated (respectively) *actin-GFP *mice. Representative photomicrographs of WT PMECs ingesting GFP+ apoptotic thymocytes are shown. **D**. Flow cytometric analysis of WT and *mertk^-/- ^*PMECs incubated for 4 hours in the presence of apoptotic cells (ACs) or live cells (LCs). N> 30,000 cells examined per genotype. Value in right panel indicates percentage of total cells that were GFP+. **E**. Real-time RT-PCR was used to measure mertk mRNA expression in whole mammary RNA harvested from 6 weeks virgin female mice, or from female mice at 1, 3, 10, and 60 dpfw. Values shown represent the average relative values, ± S.D. N = 3, each analyzed six times. **F**. Mammary glands harvested at 16.5 dpc and 1 dpfw were immunostained for MerTK. Representative images are shown. **G**. Real-time RT-PCR was used to measure mertk mRNA expression in total RNA harvested from WT PMECs cultured alone or for 4 hours in the presence of apoptotic thymocytes for 4 hours. Note that thymocytes were removed with three PBS washes prior to harvesting RNA from PMECs. Where indicated, PMECs were pre-incubated with an inhibitory MerTK-antibody or a control IgG (50 μg/μl). Values shown represent the average relative values, ± S.D. N = 3, each analyzed six times. Statistical significance using Student's T-test (unpaired).

To determine if MerTK mediates efferocytosis by MECs, we measured MerTK expression in mouse mammary glands at puberty (6 weeks of age), maturity/virgin (12 weeks of age), pregnancy (12 weeks of age) and lactation (13-17 weeks of age, 10 days after parturition). Using real-time RT-PCR, we found *mertk *mRNA expression in mammary glands at all stages, with highest relative levels detected during puberty and declining through pregnancy and lactation (Figure 6A). MerTK protein expression was detected by immunohistochemistry in MECs of the terminal end buds at six weeks of age, and at lower levels in the ductal epithelium at 12 weeks of age (Figure 6B).

Primary MECs (PMECs) from WT and *mertk^-/- ^*female mice were cultured in defined serum-free media in the presence of Gas6, then co-cultured in the presence of apoptotic thymocytes harvested from dexamethasone-treated mice harboring a transgene comprised of the actin promoter driving expression of green fluorescent protein (*actin-GFP *mice). After 4 hours of co-culture, PMECs were thoroughly washed prior to analysis by flow cytometry to detect GFP+ cells, indicating the ingestion of apoptotic GFP-expressing thymocytes. WT PMECs were capable of ingesting apoptotic thymocytes (Figure 6C). We found that approximately one-third of WT PMECs were GFP+ after culture with apoptotic thymocytes (Figure 6D). In contrast, co-culture of WT PMECs with live GFP+ thymocytes from untreated *actin-GFP *mice did not result in the detection of GFP+ cells, confirming the specificity of this assay for ACs. Finally, GFP+ cells were not detected in *mertk^-/- ^*PMECs cultured with apoptotic thymocytes, suggesting that MerTK is required for the phagocytosis of ACs by mammary epithelial cells.

### Apoptotic cells induce the expression of MerTK during early involution

The levels of *mertk *mRNA expression were measured during involution. Compared to what was seen in mammary glands harvested at 6 weeks of age, *mertk *was dramatically elevated nearly 24-fold by 1 dpfw (Figure 6E). These results were confirmed by immunohistochemical detection of MerTK (Figure 6F), showing a sharp induction of epithelial MerTK expression in mammary glands harvested from mice at 1 dpfw as compared to what was seen in during pregnancy (16.5 dpc). Levels of *mertk *peak at 3 dpfw, but remain elevated for at least 60 dpfw as compared to what was observed in mammary glands from virgin mice (Figure 6E). Previous reports suggest that apoptotic lung epithelial cells can induce expression of MerTK in alveolar macrophages [32]. Therefore, we tested the ability of apoptotic cells to induce *mertk *mRNA expression in cultured MECs. Compared to MECs cultured alone or ACs cultured alone, MECs cultured in the presence of ACs for 4 hours followed by removal of ACs resulted in a >11-fold induction of MerTK mRNA (Figure 6G). Interestingly, induction of *mertk *expression was partially impaired in MECs pre-incubated with an inhibitory antibody against MerTK, suggesting that induction of MerTK by ACs is controlled in a MerTK-induced positive feedback loop.

### Impaired efferocytosis alters cytokine expression and signaling at the earliest stages of involution in the mammary gland

**Figure 7:**
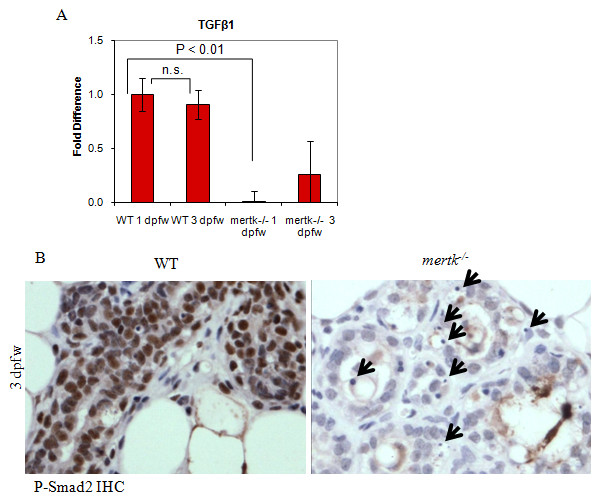
**Impaired apoptotic cell clearance during early involution results in failure to induce TGFβ1 signaling**. **A**. Real-time PCR of total RNA harvested from mammary glands of WT and *mertk^-/- ^*females at 1 and 3 dpfw. Relative values were calculated for each sample using the δδCT method described in Figure 6. Mammary RNA harvested from each mouse was analyzed. Shown is the relative average value (± S.D.) (analyzed 6 times each) setting the values obtained for WT mammary glands harvested at 1 dpfw equal to 1. N = 3. Statistical significance using Student's T-test (unpaired). **B**. Immunohistochemical detection of phospho-Smad2 in mammary glands harvested at 3 dpfw. Arrows indicate apoptotic bodies. Representative images are shown, N = 3.

Professional phagocytes secrete immunomodulatory cytokines in response to AC ingestion. We measured expression of immunomodulatory cytokines in the mammary gland using qRT-PCR on total mammary RNA harvested at 1 and 3 dpfw. Transforming growth factor β1 (TGFβ1) mRNA levels were nearly undetectable in *mertk^-/- ^*samples as compared to WT at 1 dpfw (Figure 7A). By 3 dpfw, TGFβ1 levels increased in MerTK-deficient mammary glands, but still remained below what was seen in WT mammary glands at 3 dpfw. Smad2 is a transcription factor that is phosphorylated in response to TGFβ1 signaling. P-Smad2 was abundant in epithelial nuclei of WT mammary glands at 3 dpfw, (Figure 7B). In contrast, P-smad2 was not detected in the mammary epithelium of MerTK-deficient mice at 3 dpfw. Numerous uningested apoptotic bodies were noted in the MerTK-deficient samples, however. These results demonstrate that cytokine modulation occurs in the earliest stages of involution, possibly as a result of efferocytosis, and that signaling to neighboring cells in response to cytokine modulation occurs. Furthermore, these results demonstrate that loss of MerTK-mediated efferocytosis by the mammary epithelium impairs the production of TGFβ1, a cytokine involved in dampening acute inflammation and wound healing responses. While the role of TGFβ1 in efferocytosis and early involution are currently not known, these results suggest that efferocytosis-mediated cytokine regulation may impact homeostasis and remodeling in the post-lactational mammary gland.

## Discussion

The mammary gland offers a unique model organ in which to study apoptosis on a massive yet physiologic scale, and to investigate the mechanisms involved in the clearance of ACs from the tissue environment. We present the first study illustrating the critical nature of AC clearance in preserving homeostasis during post-lactational involution, and how failure of this process contributes to sustained pathologies in the post-partum mammary gland. The consequences of impaired efferocytosis include the loss of TGFβ1 signaling, hyperproliferation, increased vascularization, fibrosis, and a decreased ability to nurse second litters due to impaired remodeling of the *mertk^-/- ^*mammary gland following lactation.

Our results suggest that during the earliest stages of involution, efferocytosis by MECs is a critical process. We used mice deficient for MerTK, a receptor tyrosine kinase with a known role in directing AC clearance in macrophages. The data showed that MerTK, but not the related TAM receptor family members Axl and Tyro3 (Figure 4), was required for AC clearance by the mammary epithelium (Figure 1 and Figure 6). Interestingly, the phenotypic effects of MerTK-deficiency were not apparent in mammary glands of virgin female mice (Figure 3), but manifested only after a single round of pregnancy, lactation, and involution, and are therefore specific to the post-partum environment. Furthermore, the pathologies evident in *mertk^-/- ^*mammary glands persisted through at least 60 dpfw (Figure 2), suggesting that the increased epithelial content observed in *mertk^-/- ^*mammary glands was not due to delayed involution, since the phenotype did not resolve even over extended time points. These observations demonstrate the critical nature of rapid AC clearance during involution to prevent tissue damage within the post-partum mammary gland.

MerTK-directed phagocytosis by non-professional phagocytes has been described previously in the pigmented epithelium of the retina, which uses MerTK and the αvβ5 integrin to engulf spent rod photoreceptor outer segments that are shed daily [24,33-38]. Spermatozoa and Sertoli cells also use MerTK to direct the phagocytosis of neighboring ACs [27,39]. Currently, there are no reports describing a role for Axl and Tyro3 in phagocytic clearance of ACs by non-professional phagocytes, although expression of each of these receptors is detected in several epithelial organs. Therefore, while each of the TAM receptors directs clearance of ACs, the cell type in which these receptors are expressed may influence their role in this process.

MECs utilize many of the same molecules for AC clearance as professional phagocytes, supporting the observation that MerTK can direct efferocytosis by MECs and macrophages (Figure 6 and [22,23,25]). In cell culture, uptake of ACs by MECs can be partially reduced by the addition of inhibitors to C1q and MBP, two collectins that target ACs for phagocytosis. Furthermore, the lipopolysaccharide receptor CD14 is an AC scavenger receptor, and is abundantly expressed in MECs, although its role in directing AC clearance by the mammary epithelium has not yet been reported [3,4]. However, this is not to say that macrophages do not play a role in AC clearance in the mammary gland during involution. Macrophage infiltration is detected between involution days 2 and 4, at which time they have been shown to engulf dying MECs and to release anti-inflammatory cytokines, such as TGFβ1. *mertk^-/- ^*macrophage have defects in phagocytosis of apoptotic targets, and studies performed *ex vivo *demonstrated that LPS-stimulated *mertk^-/- ^*macrophage produce overwhelming levels of inflammatory cytokines, and are unable to induce the expression of anti-inflammatory cytokines such as TGFβ1. At this time, the respective relative contributions of the epithelial and macrophage populations are unclear. While we have demonstrated that MerTK-expressing macrophages (and other host-derived stromal cells) are unable to compensate for the loss of MerTK expression in the mammary epithelium of WT mammary fat pads reconstituted with MerTK-deficient epithelium (Figure 5), these studies do not rule out a role for macrophages in efferocytosis during involution. Unfortunately, reciprocal transplants of WT mammary epithelium into the cleared fat pads of MerTK-deficient mice did not produce any outgrowths for analysis (0/8 mice analyzed), and demonstrated a strong inflammatory response at the transplant site, suggesting graft rejection by the host. Therefore, we were unable to assess the effects of MerTK-deficient macrophages using this model, and future studies are focused on developing tissue-specific models of MerTK-deficiency to investigate these questions.

## Conclusion

In summary, loss of AC clearance during a single cycle of lactation/involution resulted in pathologic changes that prevented subsequent lactation. Changes include increased epithelial cell proliferation vascularization, and ECM deposition. These studies highlight the importance of AC clearance in tissue homeostasis and in epithelial-stromal communications. Furthermore, these studies describe a novel role for MerTK in the mammary gland. The profound effects rendered by MerTK on mammary tissue homeostasis suggest that MerTK may be critical in other tissues that require extensive and rapid remodeling under physiologic circumstances, for example, the ovaries following the estrous cycle/menstrual cycle, or the uterus at parturition. It is also interesting to speculate about the relationship between pregnancy-associated breast cancer, the impact of involution on breast cancer malignancy, and the role that efferocytosis plays in this process.

## List of Abbreviations

AC: apoptotic cell; dpc: days post-conception; dpfw: days post-forced wean; MEC: mammary epithelial cell; PCNA: proliferating cell nuclear antigen; RTK: receptor tyrosine kinase; TAM: Tyro3/Axl/MerTK

## Competing interests

The authors declare that they have no competing interests.

## Authors' contributions

RSC designed experiments, interpreted data, and wrote the manuscript. MAS, DMH, and KES executed experiments. HSE interpreted data and provided reagents. All authors have read and approved the final manuscript.

## References

[B1] Cunha GR Hom YK Role of mesenchymal-epithelial interactions in mammary gland development J Mammary Gland Biol Neoplasia 1996 1 1 21 35 10.1007/BF02096300 10887478

[B2] Robinson GW Karpf AB Kratochwil K Regulation of mammary gland development by tissue interaction J Mammary Gland Biol Neoplasia 1999 4 1 9 19 10.1023/A:1018748418447 10219903

[B3] Stein T Morris JS Davies CR Weber-Hall SJ Duffy MA Heath VJ Bell AK Ferrier RK Sandilands GP Gusterson BA Involution of the mouse mammary gland is associated with an immune cascade and an acute-phase response, involving LBP, CD14 and STAT3 Breast Cancer Res 2004 6 2 R75 91 10.1186/bcr753 14979920 PMC400652

[B4] Atabai K Sheppard D Werb Z Roles of the innate immune system in mammary gland remodeling during involution J Mammary Gland Biol Neoplasia 2007 12 1 37 45 10.1007/s10911-007-9036-6 17286210 PMC2574498

[B5] Monks J Smith-Steinhart C Kruk ER Fadok VA Henson PM Epithelial cells remove apoptotic epithelial cells during post-lactation involution of the mouse mammary gland Biol Reprod 2008 78 4 586 594 10.1095/biolreprod.107.065045 18057312

[B6] Gouon-Evans V Rothenberg ME Pollard JW Postnatal mammary gland development requires macrophages and eosinophils Development 2000 127 11 2269 2282 10804170 10.1242/dev.127.11.2269

[B7] Van Nguyen A Pollard JW Colony stimulating factor-1 is required to recruit macrophages into the mammary gland to facilitate mammary ductal outgrowth Dev Biol 2002 247 1 11 25 10.1006/dbio.2002.0669 12074549

[B8] Wiktor-Jedrzejczak W Bartocci A Ferrante AW Ahmed-Ansari A Sell KW Pollard JW Stanley ER Total absence of colony-stimulating factor 1 in the macrophage-deficient osteopetrotic (op/op) mouse Proc Natl Acad Sci USA 1990 87 12 4828 4832 10.1073/pnas.87.12.4828 2191302 PMC54211

[B9] Pollard JW Hennighausen L Colony stimulating factor 1 is required for mammary gland development during pregnancy Proc Natl Acad Sci USA 1994 91 20 9312 9316 10.1073/pnas.91.20.9312 7937762 PMC44802

[B10] de Almeida CJ Linden R Phagocytosis of apoptotic cells: a matter of balance Cell Mol Life Sci 2005 62 14 1532 1546 10.1007/s00018-005-4511-y 15905967 PMC11139073

[B11] Humphreys RC Krajewska M Krnacik S Jaeger R Weiher H Krajewski S Reed JC Rosen JM Apoptosis in the terminal endbud of the murine mammary gland: a mechanism of ductal morphogenesis Development 1996 122 12 4013 4022 9012521 10.1242/dev.122.12.4013

[B12] Andres AC Strange R Apoptosis in the estrous and menstrual cycles J Mammary Gland Biol Neoplasia 1999 4 2 221 228 10.1023/A:1018737510695 10426401

[B13] Monks J Rosner D Geske FJ Lehman L Hanson L Neville MC Fadok VA Epithelial cells as phagocytes: apoptotic epithelial cells are engulfed by mammary alveolar epithelial cells and repress inflammatory mediator release Cell Death Differ 2005 12 2 107 114 10.1038/sj.cdd.4401517 15647754

[B14] Behrens EM Gadue P Gong SY Garrett S Stein PL Cohen PL The mer receptor tyrosine kinase: expression and function suggest a role in innate immunity Eur J Immunol 2003 33 8 2160 2167 10.1002/eji.200324076 12884290

[B15] Graham DK Bowman GW Dawson TL Stanford WL Earp HS Snodgrass HR Cloning and developmental expression analysis of the murine c-mer tyrosine kinase Oncogene 1995 10 12 2349 2359 7784083

[B16] Graham DK Dawson TL Mullaney DL Snodgrass HR Earp HS Cloning and mRNA expression analysis of a novel human protooncogene, c-mer Cell Growth Differ 1994 5 6 647 657 8086340

[B17] Ling L Kung HJ Mitogenic signals and transforming potential of Nyk, a newly identified neural cell adhesion molecule-related receptor tyrosine kinase Mol Cell Biol 1995 15 12 6582 6592 8524223 10.1128/mcb.15.12.6582 PMC230911

[B18] Lai C Gore M Lemke G Structure, expression, and activity of Tyro 3, a neural adhesion-related receptor tyrosine kinase Oncogene 1994 9 9 2567 2578 8058320

[B19] Caraux A Lu Q Fernandez N Riou S Di Santo JP Raulet DH Lemke G Roth C Natural killer cell differentiation driven by Tyro3 receptor tyrosine kinases Nat Immunol 2006 7 7 747 754 10.1038/ni1353 16751775

[B20] Lemke G Lu Q Macrophage regulation by Tyro 3 family receptors Curr Opin Immunol 2003 15 1 31 36 10.1016/S0952-7915(02)00016-X 12495730

[B21] Lu Q Lemke G Homeostatic regulation of the immune system by receptor tyrosine kinases of the Tyro 3 family Science 2001 293 5528 306 311 10.1126/science.1061663 11452127

[B22] Seitz HM Camenisch TD Lemke G Earp HS Matsushima GK Macrophages and dendritic cells use different Axl/Mertk/Tyro3 receptors in clearance of apoptotic cells J Immunol 2007 178 9 5635 5642 17442946 10.4049/jimmunol.178.9.5635

[B23] Cohen PL Caricchio R Abraham V Camenisch TD Jennette JC Roubey RA Earp HS Matsushima G Reap EA Delayed apoptotic cell clearance and lupus-like autoimmunity in mice lacking the c-mer membrane tyrosine kinase J Exp Med 2002 196 1 135 140 10.1084/jem.20012094 12093878 PMC2194017

[B24] Duncan JL LaVail MM Yasumura D Matthes MT Yang H Trautmann N Chappelow AV Feng W Earp HS Matsushima GK An RCS-like retinal dystrophy phenotype in mer knockout mice Invest Ophthalmol Vis Sci 2003 44 2 826 838 10.1167/iovs.02-0438 12556419

[B25] Scott RS McMahon EJ Pop SM Reap EA Caricchio R Cohen PL Earp HS Matsushima GK Phagocytosis and clearance of apoptotic cells is mediated by MER Nature 2001 411 6834 207 211 10.1038/35075603 11346799

[B26] Sen P Wallet MA Yi Z Huang Y Henderson M Mathews CE Earp HS Matsushima G Baldwin AS Tisch RM Apoptotic cells induce Mer tyrosine kinase-dependent blockade of NF-kappaB activation in dendritic cells Blood 2007 109 2 653 660 10.1182/blood-2006-04-017368 17008547 PMC1785106

[B27] Lu Q Gore M Zhang Q Camenisch T Boast S Casagranda F Lai C Skinner MK Klein R Matsushima GK Tyro-3 family receptors are essential regulators of mammalian spermatogenesis Nature 1999 398 6729 723 728 10.1038/19554 10227296

[B28] Muraoka RS Lenferink AE Simpson J Brantley DM Roebuck LR Yakes FM Arteaga CL Cyclin-dependent kinase inhibitor p27(Kip1) is required for mouse mammary gland morphogenesis and function J Cell Biol 2001 153 5 917 932 10.1083/jcb.153.5.917 11381079 PMC2174338

[B29] Muraoka RS Lenferink AE Law B Hamilton E Brantley DM Roebuck LR Arteaga CL ErbB2/Neu-induced, cyclin D1-dependent transformation is accelerated in p27-haploinsufficient mammary epithelial cells but impaired in p27-null cells Mol Cell Biol 2002 22 7 2204 2219 10.1128/MCB.22.7.2204-2219.2002 11884607 PMC133673

[B30] Lemke G Burstyn-Cohen T TAM receptors and the clearance of apoptotic cells Ann N Y Acad Sci 2010 1209 23 29 10.1111/j.1749-6632.2010.05744.x 20958312 PMC3061224

[B31] Lemke G Rothlin CV Immunobiology of the TAM receptors Nat Rev Immunol 2008 8 5 327 336 10.1038/nri2303 18421305 PMC2856445

[B32] Kazeros A Harvey BG Carolan BJ Vanni H Krause A Crystal RG Overexpression of apoptotic cell removal receptor MERTK in alveolar macrophages of cigarette smokers Am J Respir Cell Mol Biol 2008 39 6 747 757 10.1165/rcmb.2007-0306OC 18587056 PMC2586050

[B33] Duncan JL Yang H Vollrath D Yasumura D Matthes MT Trautmann N Chappelow AV Feng W Earp HS Matsushima GK Inherited retinal dystrophy in Mer knockout mice Adv Exp Med Biol 2003 533 165 172 15180261 10.1007/978-1-4615-0067-4_21

[B34] Gal A Li Y Thompson DA Weir J Orth U Jacobson SG Apfelstedt-Sylla E Vollrath D Mutations in MERTK, the human orthologue of the RCS rat retinal dystrophy gene, cause retinitis pigmentosa Nat Genet 2000 26 3 270 271 10.1038/81555 11062461

[B35] Prasad D Rothlin CV Burrola P Burstyn-Cohen T Lu Q Garcia de Frutos P Lemke G TAM receptor function in the retinal pigment epithelium Mol Cell Neurosci 2006 33 1 96 108 10.1016/j.mcn.2006.06.011 16901715

[B36] Finnemann SC Nandrot EF MerTK activation during RPE phagocytosis in vivo requires alphaVbeta5 integrin Adv Exp Med Biol 2006 572 499 503 full_text 17249615 10.1007/0-387-32442-9_69 PMC3577060

[B37] Nandrot EF Anand M Almeida D Atabai K Sheppard D Finnemann SC Essential role for MFG-E8 as ligand for alphavbeta5 integrin in diurnal retinal phagocytosis Proc Natl Acad Sci USA 2007 104 29 12005 12010 10.1073/pnas.0704756104 17620600 PMC1924559

[B38] Nandrot EF Kim Y Brodie SE Huang X Sheppard D Finnemann SC Loss of synchronized retinal phagocytosis and age-related blindness in mice lacking alphavbeta5 integrin J Exp Med 2004 200 12 1539 1545 10.1084/jem.20041447 15596525 PMC2211990

[B39] Xiong W Chen Y Wang H Wu H Lu Q Han D Gas6 and the Tyro 3 receptor tyrosine kinase subfamily regulate the phagocytic function of Sertoli cells Reproduction 2008 135 1 77 87 10.1530/REP-07-0287 18159085

